# Surgical Training Pathways in the United Kingdom and the United States: Lessons for Resource-Limited Settings From High-Income Countries

**DOI:** 10.7759/cureus.73285

**Published:** 2024-11-08

**Authors:** Adeel Nadeem

**Affiliations:** 1 Orthopaedics, Royal National Orthopaedic Hospital, London, GBR

**Keywords:** competence-based training, health disparity, postgraduate surgical training, resource limited, resource-limited setting, surgical training programme, trainee well-being

## Abstract

Background

Surgical training is a critical component of healthcare, especially in high-income countries such as the United Kingdom (UK) and the United States (US), which have established distinct, well-organised training frameworks. Comparing these systems provides valuable insights that may enhance global surgical education, particularly in low- and middle-income countries, where training and retaining proficient surgeons are considerable challenges.

Methodology

This comparative study examines the surgical training systems in the UK and the US, focusing on key aspects, including training structure, competency-based assessments, and work-hour regulations. Data were sourced from regulatory organisations such as the General Medical Council, the Royal College of Surgeons, the Accreditation Council for Graduate Medical Education, and the American Board of Surgery. The analysis explores how elements of these models might be adapted to support sustainable surgical education frameworks in resource-limited environments. Ethical approval was not required due to the use of publicly accessible data and no patient involvement.

Results

The UK and US surgical training systems differ substantially in their structure, training duration, and specialisation timing. The UK employs a tiered approach, offering generalist experience before specialisation, while the US favours early specialisation directly after medical school. Both systems implement competency-based evaluations, though the US system places a greater emphasis on case volume and procedural exposure. Work-hour regulations also vary, with the UK capping weekly hours at 48 under the European Working Time Directive, compared to an 80-hour maximum in the US, which results in differing levels of trainee satisfaction and burnout rates.

Conclusions

The competency-based assessments in both the UK and the US offer adaptable frameworks for resource-limited settings. The phased training approach in the UK is well-suited for environments requiring versatile surgeons capable of handling a wide range of cases. By implementing these adaptable elements, along with cost-effective training innovations such as simulation tools, e-learning platforms, and international partnerships, resource-constrained regions can foster a sustainable, skilled surgical workforce. These insights offer pathways to improve healthcare outcomes and equity globally by enhancing surgical capacity in regions with limited resources.

## Introduction

Surgical training is an essential element of healthcare systems, particularly in high-income countries such as the United Kingdom (UK) and the United States (US). These nations have established extensive training frameworks that differ in structure, length, and evaluation techniques. Understanding these distinctions is crucial for enhancing international medical education and for drawing insights relevant to resource-limited environments, where medical facilities and access to skilled surgeons are frequently limited.

Approximately 5 billion individuals worldwide lack access to safe and affordable surgical treatment, predominantly in low- and middle-income countries (LMICs) [1]. Significant shortages of qualified surgeons and disparities in access to surgical interventions continue to exist in these areas. This article analyses the fundamental components of the surgical training systems in the UK and the US, specifically entry requirements, training timelines, work hours, and assessments, to discover transferable solutions for enhancing sustainable surgical capacity in resource-constrained environments. This work contributes to global discussions on enhancing surgical education and improving healthcare delivery in resource-limited settings.

## Materials and methods

This comparative analysis reviewed publicly accessible data from regulatory organisations, including the General Medical Council (GMC), the Royal College of Surgeons (RCS), the Intercollegiate Surgical Curriculum Programme (ISCP), the Accreditation Council for Graduate Medical Education (ACGME), and the American Board of Surgery (ABS). The evaluation encompassed essential elements of trainee well-being, competency-based assessments, work-hour regulations and trainee satisfaction. Global health data from resource-constrained settings were examined to assess the potential adaptation of components from the surgical training systems of the UK and the US to these areas. This study did not involve human subjects, thus ethical approval was not required.

## Results

Entry requirements, training duration, and structure

The surgical training programs in the UK and the US differ considerably regarding entry requirements, organisational framework, and length of training. In the UK, medical graduates initially complete two years of Foundation Training (FT), during which they acquire experience in many medical specialities, including surgery. Upon finishing FT, individuals enrol for Core Surgical Training (CST), a two-year curriculum that offers generalised surgical experience. After completing CST, trainees advance to Specialty Training (ST), which spans six to eight years, depending upon the selected surgical specialty [2].

Conversely, the US system adopts a more straightforward approach. Following medical school, graduates directly apply to surgical residency programs via the National Resident Matching Program (NRMP). Residency spans five to seven years, during which trainees specialise early in their selected discipline. Fellowship programs provide an additional one to three years of sub-specialised training [3]. The dependence on the NRMP’s matching mechanism establishes a competitive yet effective route into surgical residency.

**Figure 1:**
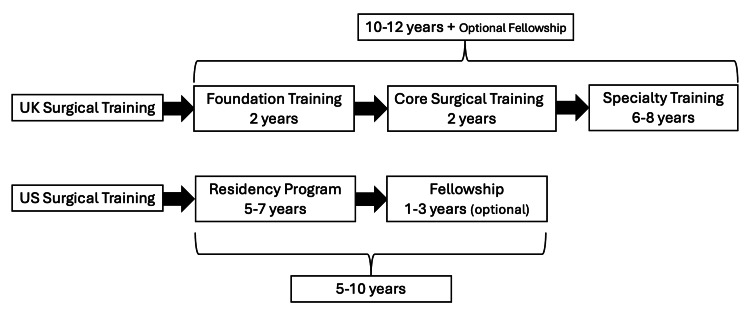
Surgical training structure comparison. References [2,3].

The disparities in training structure underscore contrasting philosophies towards specialisation, highlighted in Figure 1. The UK’s tiered approach permits students to investigate multiple surgical disciplines before selecting a specialty, yielding doctors with extensive and varied experience [4]. However, the protracted training duration postpones the commencement of full clinical practice. In contrast, the US system emphasises early specialisation, allowing trainees to acquire extensive expertise in their selected discipline more rapidly, with the majority of surgeons finishing primary training earlier. Subspecialty fellowships may prolong overall training duration, although they guarantee that US surgeons commence practice with an elevated level of specialised expertise [5].

Competency-based assessments

The UK and the US have implemented competency-based frameworks for surgical education. These evaluations are crucial for guaranteeing that students acquire the necessary abilities to perform intricate surgical procedures. The ISCP in the UK underpins competency assessment through organised annual evaluations termed the Annual Review of Competence Progression (ARCP). This framework underscores the advancement of abilities through practical experience and formal examinations, including the Membership of the Royal College of Surgeons (MRCS) and Fellowship of the Royal College of Surgeons (FRCS), which are requisite for certification [6].

In the US, competency-based evaluations are incorporated into the ACGME’s milestone framework, which evaluates residents on particular clinical and procedural competencies during their training. Furthermore, US surgical residents maintain detailed logs of their operating experiences, which are routinely evaluated to confirm their compliance with the requisite procedural competencies [5,7]. Both systems ensure that trainees meet high standards of competence before becoming certified surgeons; however, the US system prioritises case volume and procedural experience to a higher extent.

Work hours and trainee welfare

Regulations governing work hours vary considerably between the UK and US systems, affecting both trainee welfare and operational exposure. The United Kingdom adheres to the European Working Time Directive (EWTD), which limits working hours to a maximum of 48 per week. This policy aims to enhance work-life equilibrium and mitigate burnout among trainees. Concerns have arisen that reduced hours may lead to diminished operative opportunities, thereby limiting clinical competence upon certification [8].

In the US, the ACGME permits residents to work a maximum of 80 hours per week. This law, while exceeding the UK’s maximum, guarantees that residents acquire greater practical experience during their training. Extended working hours correlate with elevated burnout rates and diminished well-being, with literature quoting that over 40% of US surgical residents exhibit burnout symptoms, in contrast to 24% in the UK [9,10].

Satisfaction among surgical trainees in the UK and the US is influenced by factors such as work hours, training duration, and the competitiveness of securing preferred training positions. Trainees in the UK typically express greater satisfaction owing to organised training programs and controlled working hours in accordance with the EWTD. The extended duration of training and the competitive aspects of entering ST are significant sources of frustration [10].

Trainee satisfaction in the US is more varied. Many residents value the concentrated emphasis on surgical training, facilitating early specialisation and swift integration into their selected discipline. Nonetheless, concerns regarding the financial strain of medical education, extensive working hours, and intense pressure to obtain competitive jobs detract from overall satisfaction [11]. Moreover, the elevated incidence of burnout among US residents, partially due to demanding schedules, has been associated with reduced satisfaction levels [12].

Burnout is a considerable concern for trainees in both systems; however, the extent and nature of the issue differ. In the UK, despite reduced working hours, burnout persists as an issue, but at a lower incidence than in the US. The longer duration of UK training, however, creates a different form of stress, trainees often express frustration at the drawn-out timeline to achieving full consultant status (independent practitioner - US Attendant equivalent).

The findings of this comparative analysis have been summarised in Table 1.

**Table 1:** Key parameters of surgical training between the UK and the US. Adapted from references: [2-12].

Parameter	UK surgical training	US surgical training
Entry requirements	Medical degree (5–6 years) + 2-year Foundation Programme; competitive application to Core Surgical Training or directly to Specialty Training after foundation	Medical degree (4 years) following undergraduate degree; competitive application to surgical residency after passing the United States Medical Licensing Examination (USMLE) Step 1 and 2 exams
Training duration	10–12+ years post-medical school: Foundation (2 years) + Core Surgical Training (2 years) + Specialty Training (6+ years depending on specialty).	5–7 years post-medical school in residency, with additional 1–3 years for fellowship if subspecialising
Specialisation timing	After 2 years of Core Surgical Training, trainees apply for Specialty Training positions	Specialisation determined at the time of matching into residency, with further subspecialisation options during fellowship after residency
Competency assessments	Primarily competency-based; assessed through the Intercollegiate Surgical Curriculum Programme (ISCP) portfolio and Workplace-Based Assessments	Competency is evaluated through milestones and feedback throughout residency; semi-annual reviews and Accreditation Council for Graduate Medical Education (ACGME) requirements are mandatory
Examinations	Membership of the Royal Colleges of Surgeons (MRCS) after Core Training; Fellowship of the Royal Colleges of Surgeons (FRCS) during specialty training	USMLE Steps 1, 2, and 3; board certification exams after residency and fellowship if subspecialising
Work-hours regulation	Limited to 48 hours per week under the European Working Time Directive (EWTD), though exceptions are made	Limited to 80 hours per week by the ACGME; no more than 24 hours continuously
Trainee well-being	Lower burnout rates (24%); improved work-life balance but concerns over fewer operative opportunities. Well-being programs vary by hospital; recently, there’s an increasing focus on mental health support and work-life balance initiatives	Higher burnout rates (40%); greater operative experience but with work-life balance challenges. Hospitals offer wellness resources; focus on burnout prevention, but support varies by institution. Increasing availability of mental health resources

## Discussion

The comparison of surgical training pathways in the UK and the US highlights multiple lessons that can be applied in resource-limited environments. These regions, typically marked by considerable healthcare staff deficiencies and inadequate infrastructure, can gain advantages from elements of both training systems. The objective is to establish sustainable surgical education frameworks that reconcile quality, accessibility, and resource constraints.

Adaptability in training framework

The UK’s phased training structure, which facilitates extensive experience before specialisation, might be particularly beneficial in resource-constrained environments. Many LMICs often experience a deficiency of highly specialised surgeons. A flexible, generalist approach, where trainees gain experience in various surgical fields before narrowing their focus, can ensure that surgeons are equipped to handle a wide range of cases. This adaptability is crucial in areas where specialists are scarce, and surgeons are required to perform multiple types of surgeries to meet the local population’s needs [13].

This strategy guarantees that the healthcare staff can meet urgent surgical demands while allowing for future specialisation as infrastructure and resources evolve. The phased UK approach can thus function as a framework for developing flexible surgical capacity in LMICs.

Competency-based assessments

Competency-based assessment models from the UK and the US can be modified for implementation in resource-limited environments. Practical assessments, which prioritise experiential skills and real-world procedural competence, are especially crucial in fields where traditional theoretical education may be limited. The emphasis on milestone-based evaluations in the US system, along with the utilisation of case logs to monitor procedural experience, presents a model that can be implemented with limited infrastructure.

In resource-constrained environments, formal examinations may be impractical due to logistical constraints; nevertheless, the implementation of mentorship-based evaluations and practical competency assessments can guarantee that surgeons receive sufficient training. This can help alleviate the hazards linked to surgical migration (the “brain drain”) by ensuring that locally trained individuals possess globally recognised competencies [14].

Work hours and trainee welfare

In resource-constrained environments, surgeons frequently encounter excessive workloads resulting from significant staffing deficiencies. It is essential to adopt work-hour laws akin to those in the UK, especially in resource-limited settings, to safeguard surgeons’ well-being and enhance patient outcomes [15]. Despite limited resources, policies that prioritise surgeon well-being, such as ensuring reasonable work hours, sufficient rest, and mental health support, are crucial for the long-term retention of healthcare workers.

The use of task-sharing systems, in which mid-level health practitioners oversee non-surgical tasks, has shown promise in reducing the workload of healthcare professionals in LMICs across other medical specialities [16]. While task-sharing may not entirely resolve surgical burnout, it can alleviate some workload, enhancing rest and promoting a multidisciplinary approach to patient care.

Economical training innovations

In resource-constrained environments, the development of cost-effective technology presents a significant opportunity to improve surgical education via new teaching techniques. The implementation of economical surgical simulators, digital learning platforms, and telemedicine for remote mentorship offers an effective means to reduce the financial barriers commonly associated with traditional surgical training. E-learning and distance training programs, particularly in regions with limited access to physical training facilities, have proven to be excellent alternatives to traditional approaches [17].

Collaborative partnerships with international organisations and academic institutions based in high-income countries provide further support for bridging the surgical training gap in LMICs. These relationships are crucial for strengthening local capacity and upholding essential training standards by sharing educational materials, delivering remote mentorship, and enabling international exchanges. Programs such as the Global Surgery Initiative, which links academic institutions from affluent countries with hospitals in LMICs, have demonstrated efficacy in delivering surgical education to underserved regions, thereby fostering sustainable improvements in surgical care provision [18].

## Conclusions

The phased training structure in the UK and competency-based assessments in both the UK and the US provide adaptable frameworks that can help build sustainable surgical capacity in resource-limited settings. The UK’s generalist approach before specialisation is particularly well-suited to contexts that lack the resources for early specialisation, allowing surgeons to address diverse healthcare needs. Meanwhile, competency-based assessments from both countries ensure surgeons are trained to high standards, even in resource-constrained environments. By adopting cost-effective training innovations, such as low-cost simulation tools, e-learning platforms, and international collaborations, these settings can overcome infrastructure and educational barriers to develop a robust, skilled surgical workforce. Ultimately, these insights from the UK and the US training models offer resource-limited regions a pathway to strengthen surgical capacity and improve healthcare equity on a global scale.

## References

[1] Meara JG, Greenberg SL (2015). The Lancet Commission on Global Surgery Global surgery 2030: evidence and solutions for achieving health, welfare and economic development. Surgery.

[2] Royal College of Surgeons (2024 (2024). Surgical Curriculum - Royal College of Surgeons of England. https://www.rcseng.ac.uk/education-and-exams/curriculum.

[3] (2024). National Resident Matching Program (NRMP). Intro to the Match. https://www.nrmp.org/intro-to-the-match/.

[4] General Medical Council (2017 (2024). Designing and maintaining postgraduate assessment programmes - General Medical Council (GMC). https://www.gmc-uk.org/education/standards-guidance-and-curricula/guidance/designing-and-maintaining-postgraduate-assessment-programmes.

[5] (2024). Surgery Residency Requirements. Accreditation Council for Graduate Medical Education (ACGME). https://www.acgme.org/specialties/surgery/program-requirements-and-faqs-and-applications/.

[6] (2024). Overview of the assessment system. Intercollegiate Surgical Curriculum Programme (ISCP). https://www.iscp.ac.uk/curriculum/surgical/assessment.aspx.

[7] American Board of Surgery (ABS) (2023 (2024). General Surgery Certification - American Board of Surgery (ABS). https://www.absurgery.org/get-certified/general-surgery/.

[8] (2024). Doctors and the European Working Time Directive. British Medical Association (BMA). https://www.bma.org.uk/pay-and-contracts/working-hours/european-working-time-directive-ewtd/doctors-and-the-european-working-time-directive.

[9] Hu YY, Ellis RJ, Hewitt DB (2019). Discrimination, abuse, harassment, and burnout in surgical residency training. N Engl J Med.

[10] General Medical Council. (2024 (2024). National Training Survey 2024 - General Medical Council (GMC). National Training Survey.

[11] Kejela S, Tiruneh AG (2022). Determinants of satisfaction and self-perceived proficiency of trainees in surgical residency programs at a single institution. BMC Med Educ.

[12] Appelbaum NP, Lee N, Amendola M, Dodson K, Kaplan B (2019). Surgical resident burnout and job satisfaction: the role of workplace climate and perceived support. J Surg Res.

[13] World Health Organization. (2019 (2024). Global strategy on human resources for health: Workforce 2030. World Health Organization (WHO). https://www.who.int/publications/i/item/9789241511131.

[14] Awuah WA, Tan JK, Bharadwaj HR (2024). Surgical mentorship in low-resource environments: opportunities and challenges, a perspective. Health Sci Rep.

[15] Vitous CA, Dinh DQ, Jafri SM, Bennett OM, MacEachern M, Suwanabol PA (2021). Optimizing surgeon well-being: a review and synthesis of best practices. Ann Surg Open.

[16] Maria JL, Anand TN, Dona B, Prinu J, Prabhakaran D, Jeemon P (2021). Task-sharing interventions for improving control of diabetes in low-income and middle-income countries: a systematic review and meta-analysis. Lancet Glob Health.

[17] Lu JD, Cameron BH (2020). The effectiveness and challenges of e-learning in surgical training in low- and middle-income countries: a systematic review. Glob Health Annu Rev.

[18] (2024). Our purpose: facilitate the development of surgical care systems - The Global Surgery Foundation. https://www.globalsurgeryfoundation.org/about-us.

